# Differential expression of TGF-β superfamily members and role of Smad1/5/9-signalling in chondral versus endochondral chondrocyte differentiation

**DOI:** 10.1038/srep36655

**Published:** 2016-11-16

**Authors:** Verena Dexheimer, Jessica Gabler, Katharina Bomans, Tanja Sims, Georg Omlor, Wiltrud Richter

**Affiliations:** 1 Research Centre for Experimental Orthopaedics, Orthopaedic University Hospital Heidelberg, Heidelberg, Germany; 2 Department of Orthopaedic and Trauma Surgery, University Hospital Heidelberg, Heidelberg, Germany

## Abstract

Proteins of the transforming-growth-factor-β (TGF-β)-superfamily have a remarkable ability to induce cartilage and bone and the crosstalk of TGF-β - and BMP-signalling pathways appears crucial during chondrocyte development. Aim was to assess the regulation of TGF-β-superfamily members and of Smad2/3- and Smad1/5/9-signalling during endochondral *in vitro* chondrogenesis of mesenchymal stromal cells (MSC) relative to chondral redifferentiation of articular chondrocytes (AC) to adjust chondrocyte development of MSC towards a less hypertrophic phenotype. While MSC increased BMP4 and BMP7 and reduced TGFBR2 and TGFBR3-expression during chondrogenesis, an opposite regulation was observed during AC-redifferentiation. Antagonists CHRD and CHL2 rose significantly only in AC-cultures. AC showed higher initial BMP4, pSmad1/5/9 and SOX9 protein levels, a faster (re-)differentiation but a similar decline of pSmad2/3- and pSmad1/5/9-signalling versus MSC-cultures. BMP-4/7-stimulation of MSC-pellets enhanced SOX9 and accelerated ALP-induction but did not shift differentiation towards osteogenesis. Inhibition of BMP-signalling by dorsomorphin significantly reduced SOX9, raised RUNX2, maintained collagen-type-II and collagen-type-X lower and kept ALP-activity at levels reached at initiation of treatment. Conclusively, ALK1,2,3,6-signalling was essential for MSC-chondrogenesis and its prochondrogenic rather than prohypertrophic role may explain why inhibition of canonical BMP-signalling could not uncouple cartilage matrix production from hypertrophy as this was achieved with pulsed PTHrP-application.

Proteins of the transforming growth factor β (TGF-β) superfamily are a family of secreted signalling factors including TGF-β and bone morphogenetic proteins (BMPs) with a remarkable ability to induce cartilage and bone. During embryogenesis, chondrocytes develop in well-ordered phases of cell proliferation, condensation and maturation to proliferating chondroblasts synthesizing collagen type II and proteoglycans. Cells are then either maintained as mature chondrocytes in articular cartilage or undergo endochondral development becoming postmitotic hypertrophic chondrocytes. As such, cells express collagen type X and matrix metalloproteinase (MMP)-13 and mineralize the surrounding matrix by upregulation of alkaline phosphatase (ALP) activity to be finally replaced by bone. The importance of Smad-dependent TGF-β- and BMP-signalling pathways for cartilage and bone formation has been well established for decades and synergistic as well as antagonistic activities were evident dependent on the differentiation stage and the model^1^,^2^,^3^,^4^,^5^.

TGF-βs and BMPs bind to specific type II cell surface receptors to recruit the corresponding type I receptor to initiate signalling of their specific receptor-Smads. Generally TGF-βs depend on Smad2 and Smad3 while BMP-signalling depends on Smad1, Smad5 and Smad9 phosphorylation. Besides, TGF-β/BMP also signal via non-canonical pathways during chondrogenesis^6^. The capacity of TGF-βs and BMPs to bind to their multiple receptors is strongly influenced by several antagonists like NOG, CHRD, GREM and FST.

*In vivo*, BMPs are positive modulators of mesenchymal stromal cell (MSC) condensation and chondrocyte proliferation and negatively regulate chondrocyte terminal differentiation^7^,^8^. Complete loss of Smad1 and Smad5 in chondrocytes led to severe chondrodysplasia in mice^9^ demonstrating the importance of Smad1/5-signalling for chondrogenesis. The significance of TGF-β for cartilage and bone formation was demonstrated by conditional deletion of TGFBR2 in chondrocytes causing axial skeleton defects, alterations of hypertrophic differentiation in the growth plate and joint fusions in the phalanges^10^,^11^.

*In vitro* chondrogenesis of human MSC recapitulates part of the natural cascade of condensation, proliferation and differentiation of endochondral chondrocyte development^12^. Current standard *in vitro* differentiation protocols generating chondrocytes from MSC, however, lead to an unwanted hypertrophic development which resembles the formation of transient cartilage in the growth plate rather than the formation of stable articular cartilage^13^,^14^. Thus, cells upregulate hypertrophic markers like collagen type X, ALP and the terminal differentiation marker MMP13 which are not stimulated when expanded articular chondrocytes (AC) are exposed to identical conditions.

Our previous work demonstrated a cell-autonomous downregulation of PTHrP in favour of a strong upregulation of IHH after about 2 weeks of chondrogenesis in MSC but not AC^15^. We further provided evidence that pulsed exposure of MSC to PTHrP during this later stage of chondrogenesis allowed to further stimulate cartilage matrix deposition and at the same time strongly suppressed hypertrophy according to significant downregulation of ALP activity and indian hedgehog (IHH) expression^16^,^17^.

A recent report suggested that blocking of BMP-signalling during chondrogenesis of MSC from one exceptional donor may eliminate collagen type X and MMP13 from cartilage at maintained collagen type II and enhanced SOX9 mRNA expression^5^ suggesting that manipulation of BMP-signalling may also hold potential to shift MSC chondrogenesis towards a non-hypertrophic phenotype. Thus, aim of this study was to identify molecular determinants of the disparate reaction of MSC and AC to chondrogenic conditions with a focus on TGF-β -superfamily members and to explore the possibility to guide differentiation of MSC towards a non-hypertrophic phenotype by influencing BMP-signalling. We assessed how expression of growth factors of the TGF-β superfamily, their receptors and antagonists, and of pSmad2/3- and pSmad1/5/9-signalling is modulated during cell differentiation and whether influencing of BMP-signalling can improve MSC chondrogenesis by maintaining high cartilage matrix deposition under reduction of hypertrophy.

## Results

### Differential expression of members of the TGF-β-superfamily, their receptors and antagonists

**Figure 1:**
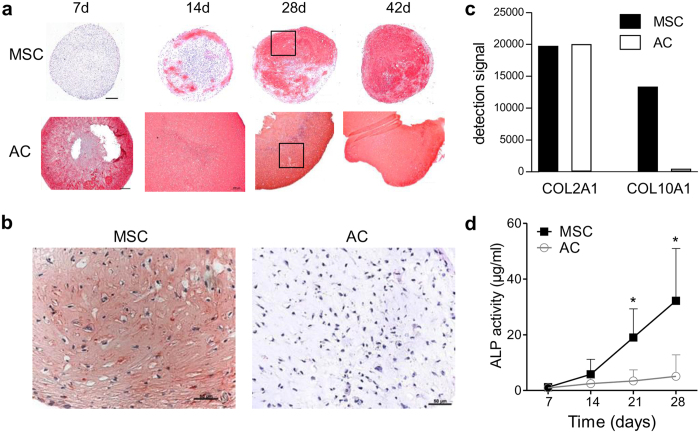
Chondrogenic differentiation of mesenchymal stromal cells (MSC) and redifferentiation of human articular chondrocytes (AC). MSC and AC pellets were cultured for 6 weeks in chondrogenic medium containing 10 ng/ml of TGF-β. (**a**) Paraffin sections were stained for collagen type II by immunohistochemistry. Representative results from one of five MSC and AC populations are shown. Scale bar: 200 μm. (**b**) Paraffin sections of day 28 pellets stained for collagen type X. Depicted are the boxed areas indicated in a. Representative results from one of five MSC and AC populations are shown. Scale bar: 50 μm. (**c**) COL2A1 and COL10A1 expression at day 28 according to microarray analysis. (**d**) Alkaline phosphatase (ALP) activity in pooled culture supernatants from 5 pellets was determined. Data are presented as mean ± SD from 5 experiments. Mann–Whitney U test: *p ≤ 0.05 versus AC at the same time points.

Under standard chondrogenic conditions, AC showed faster collagen type II and proteoglycan deposition according to histology compared to MSC-pellets (Fig. 1a) in line with higher SOX9 protein levels already at day 0 (Supplementary Fig. S1a). However, while collagen type X deposition was seen in MSC derived pellets at day 28, AC pellets remained negative throughout the culture period (Fig. 1b). Cultures from day 0 and from day 28 were subjected to transcriptome analysis and results for MSC and AC (5 donors each per group) were compared with a focus on extraction of members of the TGF-β-superfamily, their receptors and antagonists. The strong upregulation of multiple differentiation markers (Table 1) including COL2A1 confirmed successful chondrogenesis of MSC and redifferentiation of AC, respectively. Results furthermore demonstrated that AC entered into differentiation from a chondrogenic signature with higher mean SOX9 levels and expression of COL2A1 and ACAN while RUNX2 and OPN were below background (Table 1). In contrast, MSC revealed an osteochondroprogenitor cell signature according to SOX9, RUNX2 and OPN detection but absence of COL2A1 and ACAN expression. At day 28, significantly higher expression of COL10A1 was observed in MSC at day 28 at similar COL2A1 expression levels to AC cultures (Fig. 1c) which is in line with histology. Together with raising ALP activity in MSC culture supernatants (Fig. 1d) this demonstrated an endochondral differentiation exclusively in MSC.

Transcript levels of many TGF-β-superfamily growth factors were low or poorly regulated between day 0 and 28 including TGF-β1, TGF-β2 and TGF-β3 (Table 1). Interestingly, at day 0, mean expression levels of the major TGF-β and BMP factors were higher in AC compared to MSC. However, BMP2 and BMP4 showed an apparent upregulation in MSC and a downregulation during AC redifferentiation. While TGFBR2 and TGFBR3 were downregulated in MSC, they were upregulated in AC. At day 28 mean expression of the BMP subfamily antagonists FST, CHL2, CHRD and FSTL1 was higher in AC versus MSC (Table 1) with FST being downregulated during redifferentiation. NOG, CHRDL1, SOST as well as FSTL4 and 5 were below detection level in both cells types while GREM2 and FSTL1 were barely regulated. FSTL3 was upregulated in both MSC and AC. In summary, the receptors TGFBR2 and TGFBR3, the growth factors BMP2 and BMP4 and the antagonists FST, CHL2 and CHRD showed interesting regulations patterns or disparate expression between MSC and AC.

### Disparate regulation of BMP4, BMP7, TGFBR2, TGFBR3, FST, CHRD and CHL2 in MSC versus AC

**Figure 2:**
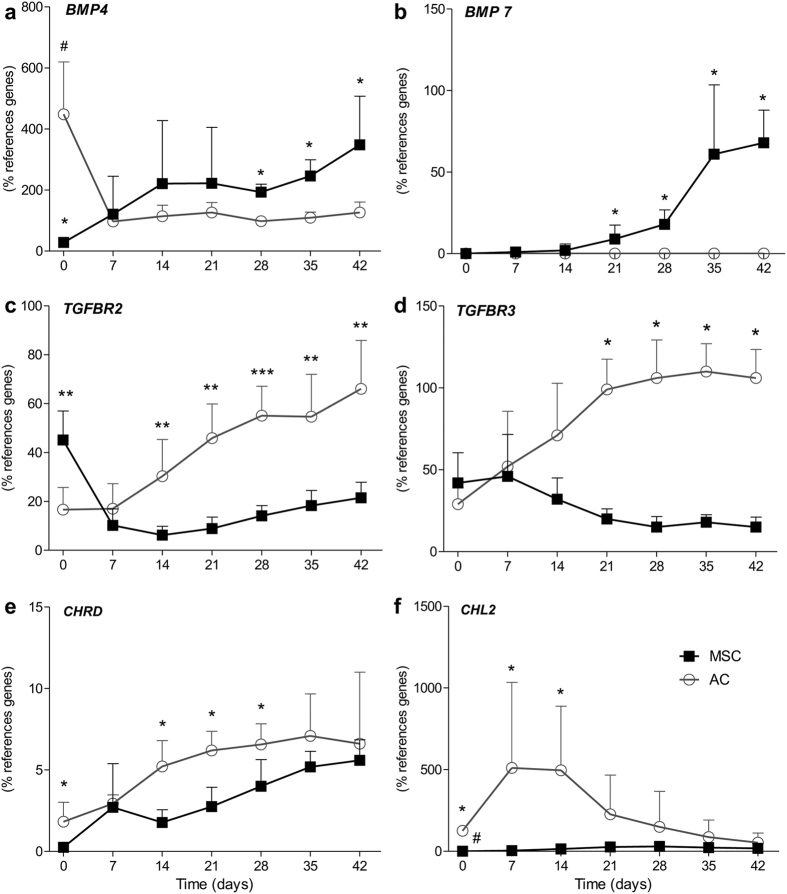
Regulation of gene expression during chondrogenic differentiation of MSC and redifferentiation of AC. MSC and AC were subjected to chondrogenic differentiation in high density pellets and 3–5 pellets per donor (n = 5) were harvested at denoted time points and pooled for RNA extraction. Gene expression was normalized to the reference genes HNRPH1 and CPSF6. Data are presented as mean ± SD of five experiments. Mann–Whitney U test: ^#^p ≤ 0.05 versus all other time points of the same cell type and *p ≤ 0.05, **p ≤ 0.01, ***p ≤ 0.001 versus the other cell type at the same time points.

Regulation of candidate molecules (bold in Table 1) was refined by qRT-PCR for a weekly time course in 5 MSC and AC donor samples each. In AC, BMP4 dropped quickly to low levels from day 0 to day 7 (Fig. 2a) and a decline of BMP6 reached significance at day 42 (data not shown). BMP7 remained negative throughout the whole culture period in AC (Fig. 2b). TGFBR2 and TGFBR3 were continuously upregulated in AC reaching significantly higher levels than in MSC at day 14 or 21 (Fig. 2c,d). BMP2 expression revealed a high donor variability in MSC, (data not shown), while BMP4 and BMP7 rose to significantly higher levels in MSC compared to AC after or at day 21 (Fig. 2a,b). In search for BMP4 and BMP7 proteins in culture supernatants and pellet lysates of AC and MSC by ELISA and Western blotting, both growth factors remained below the detection limit (data not shown) in line with their low gene expression levels.

While in MSC no significant regulation was observed for CHRD expression, this BMP antagonist raised in AC to significantly higher levels than in MSC from day 14 on (Fig. 2e). CHL2 showed a transient early upregulation only in AC and an autonomous decline later on. FST regulation resembled BMP4 regulation in AC while GREM1 showed a transient downregulation on day 7 and 14 in MSC and AC (not shown).

Thus, opposite cell-autonomous regulation of gene expression was confirmed for BMP4, BMP7, TGFBR2, TGFBR3, FST, CHRD and CHL2 in MSC versus AC. Conclusively, MSC raised molecules capable to enhance Smad1/5/9-signalling like BMP4, BMP6 and BMP7 and kept several BMP antagonists lower. Instead AC strongly downregulated BMP4, raised TGFBR2 and TGFBR3 levels and elevated antagonists CHRD and CHL2, suggesting that these differences may lead to differential pSmad-signalling in MSC versus AC.

### pSmad-signalling in MSC and AC

**Figure 3:**
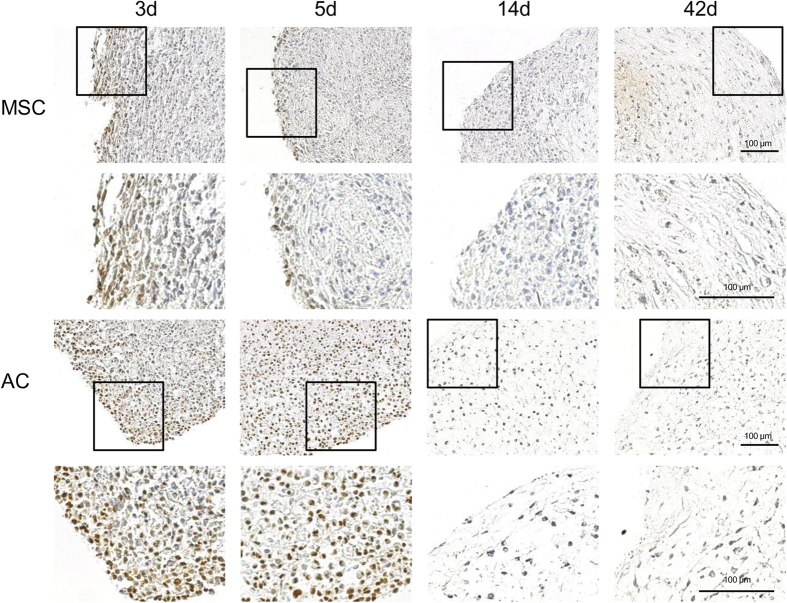
phospho-Smad1/5/9 in MSC and AC. MSC and AC pellet cultures were subjected to chondrogenic differentiation for 6 weeks. At denoted time points pellets were harvested and phospho-Smad1/5/9 was detected in paraffin-embedded sections by immunohistochemistry.

When BMP-dependent pSmad1/5/9-signalling was assessed over time in MSC versus AC cultures by histology, strongest signals were obtained in early AC pellets (Fig. 3) in line with the high BMP4 expression at initiation of culture (0d, Fig. 2a). MSC-pellets at day 3 and day 5 showed also some Smad1/5/9 phosphorylation especially in the periphery, where ALP activity usually develops later on^18^. Despite upregulation of BMP4, BMP6 and BMP7 mRNA in MSC at later stages of chondrogenesis, no net increase of pSmad1/5/9-staining was evident. Rather, a similar decline of pSmad1/5/9 staining was obvious for AC and MSC cultures in response to the continuous long-term exposure to chondrogenic medium (Fig. 3).

**Figure 4:**
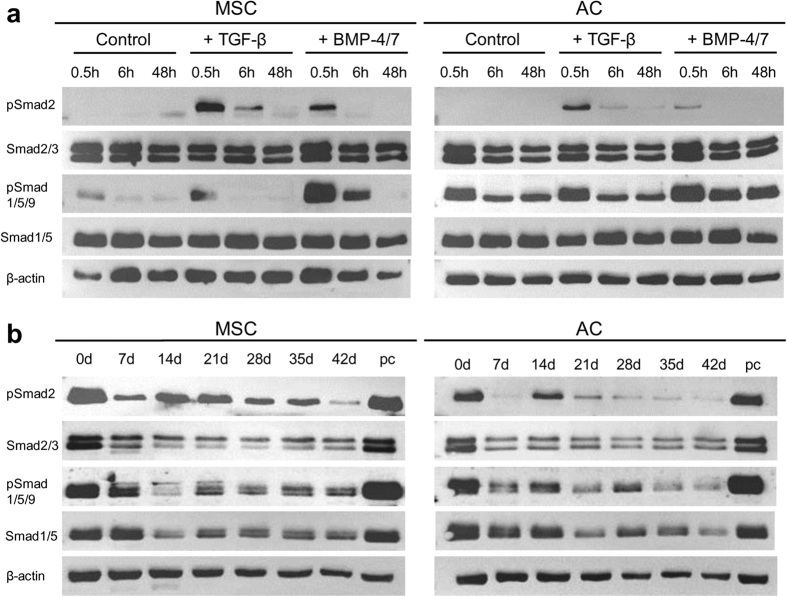
Response of MSC and AC to TGF-β and BMP-4/7. (**a**) MSC and AC were expanded up to passage 3. Pellets consisting of 5 × 10^5^ cells were exposed to basal medium (control) or basal medium supplemented with 10 ng/ml TGF-β or 100 ng/ml BMP-4/7. Three pellets per group and time point were pooled and subjected to Western blot analysis to detect phosphorylated Smad2 (pSmad2) followed by total Smad2/3 on the same blot or phosphorylated Smad1/5/9 (pSmad1/5/9) followed by total Smad1/5 on the same blot. Blots were cut in order to display β-actin on the same blot as loading control. Representative pictures of one of three independent experiments are shown. (**b**) MSC and AC pellets were exposed to chondrogenic medium (10 ng/ml TGF-β) and harvested at indicated time points. Three pellets per group and time point were pooled for Western blot analysis. Representative pictures of one of three independent experiments are shown.

Western blotting demonstrated that, at initiation of pellet culture, MSC and AC responded to TGF-β stimulation by transient elevation of pSmad2/3-signalling and to BMP-4/7 exposure by transient elevation of pSmad1/5/9-signalling as expected (Fig. 4a). TGF-β-induced pSmad2/3 signals, however, lasted longer in MSC than in AC (Fig. 4a). Interestingly, in the first 48 hours of pellet culture, AC showed constitutive pSmad1/5/9-signalling independent of growth factor treatment demonstrating a strong cell-autonomous BMP-signalling in line with higher BMP4 expression. TGF-β, however, also initiated some pSmad1/5/9-signalling in MSC and AC, just like BMP-4/7 induced some pSmad2/3-signalling emphasizing that there is significant interaction between the receptors for TGF-β and BMPs with both groups of Smads in line with findings on several other cell types^19^,^20^.

Western blot assessment of pSmad2/3- and pSmad1/5/9-signalling during weekly time intervals of MSC chondrogenesis versus AC redifferentiation revealed strikingly similar levels of pSmad2/3 and pSmad1/5/9 as well as of total Smad2/3 and Smad1/5 protein levels. Again, net pSmad1/5/9-signalling declined over time in culture in both cell types in line with the histological staining (Fig. 4b) providing no evidence for enhanced pSmad1/5/9 signals in relation to MSC hypertrophy as suggested by^5^. This result was seen when samples were harvested for Western blot analysis 48 hours after stimulation with fresh medium (3 experiments, 3 donors; Fig. 4b) or only 2 hours after stimulation with fresh chondrogenic medium (3 experiments with 3 different donors; data not shown), respectively.

### Functional relevance of BMP4 and BMP7 for chondrogenesis

In order to intensify effects of cell-autonomous upregulation of BMP4 and BMP7 during MSC chondrogenesis, exogenous BMP-4/7 heterodimer was added to chondrogenic medium from day 14 on when endogenous BMP-4/7 upregulation became apparent.

**Figure 5:**
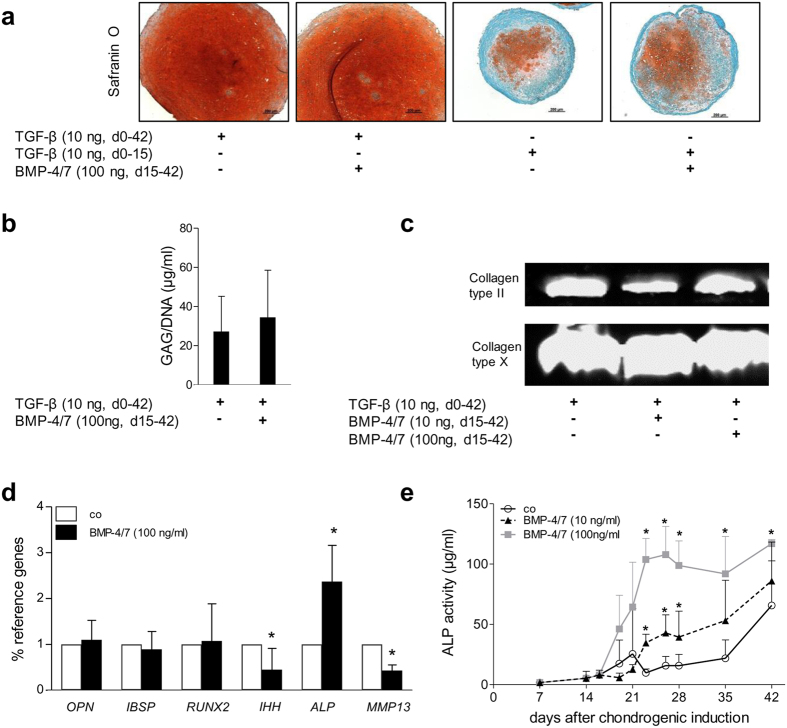
Effect of BMP-4/7 treatment on chondrogenic differentiation of MSC. (**a**) MSC (n = 5 donors) were expanded up to passage 3 and pellets consisting of 5 × 10^5^ cells were cultivated in chondrogenic medium with growth factors as indicated. Histological detection of proteoglycan deposition by Safranin O staining for one representative donor per group is shown. (**b**) Quantification of GAG per DNA. Values are shown as mean ± SD of 5 independent experiments. (**c**) Western blot analysis of collagen type II and collagen type X deposition. Collagens were extracted from 1 pellet per group on day 42. Representative results from one of three experiments shown. By cutting the blot, collagen type II and collagen type X content of the same pellet is visualized. Full-length blots are presented in Supplementary Fig. S2. (**d**) Gene expression analysis of 5 pooled pellets per MSC population and group at day 42. Gene expression was normalized to the reference genes HNRPH1 and CPSF6. Data are presented as mean ± SD (n = 5). Mann–Whitney U test, *p ≤ 0.05 versus control (co). (**e**) Culture supernatants of 5 pellets per donor, group and time point were pooled, and ALP activity in culture supernatants was determined. Data are presented as mean ± SD (n = 5 experiments). Mann–Whitney U test, *p ≤ 0.05 versus control group at the same time point.

Although BMP-4/7 heterodimer strongly stimulated cartilage matrix deposition in chondrocytes in a previous study^21^, BMP-4/7 heterodimer neither enhanced proteoglycan deposition in MSC-derived pellets relative to TGF-β alone (Fig. 5a,b), nor did it stimulate collagen type II and/or collagen type X deposition in day 42 pellets according to Western blotting (Fig. 5c). When TGF-β was withdrawn from the culture from day 14 on and instead replaced by 100 ng/ml BMP-4/7 or no other factor, cartilage matrix deposition apparently ceased in both groups (Fig. 5a). This underlined the necessity and potency of TGF-β to fully stimulate MSC chondrogenesis and the inability of BMP-4/7 to replace it.

Importantly, in the presence of TGF-β, BMP-4/7 supplementation from day 14 on also did not shift differentiation of MSC towards the osteogenic lineage according to expression of typical osteogenic markers like OPN, IBSP and RUNX2 (Fig. 5d). IHH and the terminal differentiation marker MMP13 were even significantly suppressed. Only ALP mRNA levels were significantly upregulated (Fig. 5d) and ALP activity raised more rapidly in a dose-dependent manner (Fig. 5e). In summary, in the presence of TGF-β and dexamethasone contained in standard chondrogenic medium, exogenous BMP-4/7 from day 14 did not produce a lineage shift. Accelerated ALP induction suggested, however, that cells underwent faster maturation within the endochondral lineage under stimulation with BMP4/7.

### Inhibition of Smad1/5/9-signalling by dorsomorphin

In order to further dissect the functional role of Smad1/5/9-signalling for chondrogenic differentiation and hypertrophy, chondrogenic MSC cultures were treated with the small molecule inhibitor dorsomorphin (DM) capable to block pSmad1/5/9-signalling via the ALK 1, 2, 3 and 6 receptors^22^. DM should, thus, mimic part of the combined actions of natural BMP antagonists like CHRD, and CHL2 which were upregulated by AC under chondrogenic conditions (Fig. 2e,f). When differentiating MSC pellets were exposed to 10 μM DM from day 0, no chondrogenesis occurred according to histology on day 42 (data not shown). DM treatment of MSC for the first 4 days of pellet culture reduced pSmad1/5/9 in standard chondrogenic medium and even more when cells were stimulated with BMP-4/7 instead of TGF-β, or with both factors, confirming the activity of DM on Smad1/5/9-signalling (not shown). In parallel, the Sox9 protein levels dropped considerably under DM treatment in either condition (Supplementary Fig. S1b) demonstrating a contribution of ALK1,2,3,6-signalling to early SOX9 induction. Remarkably, in the presence of DM, the additive effects of TGF-β and BMP-4/7 on SOX9 induction were eliminated and the BMP-4/7-supported SOX9 induction was even suppressed below levels of growth factor-free control cultures indicating a contribution by endogenous ALK1,2,3,6-signalling to SOX9 induction. Altogether this provided the likely explanation that chondrogenesis failed due to insufficient SOX9 induction in the continuous presence of the ALK1,2,3,6 receptor inhibitor DM from day 0.

**Figure 6:**
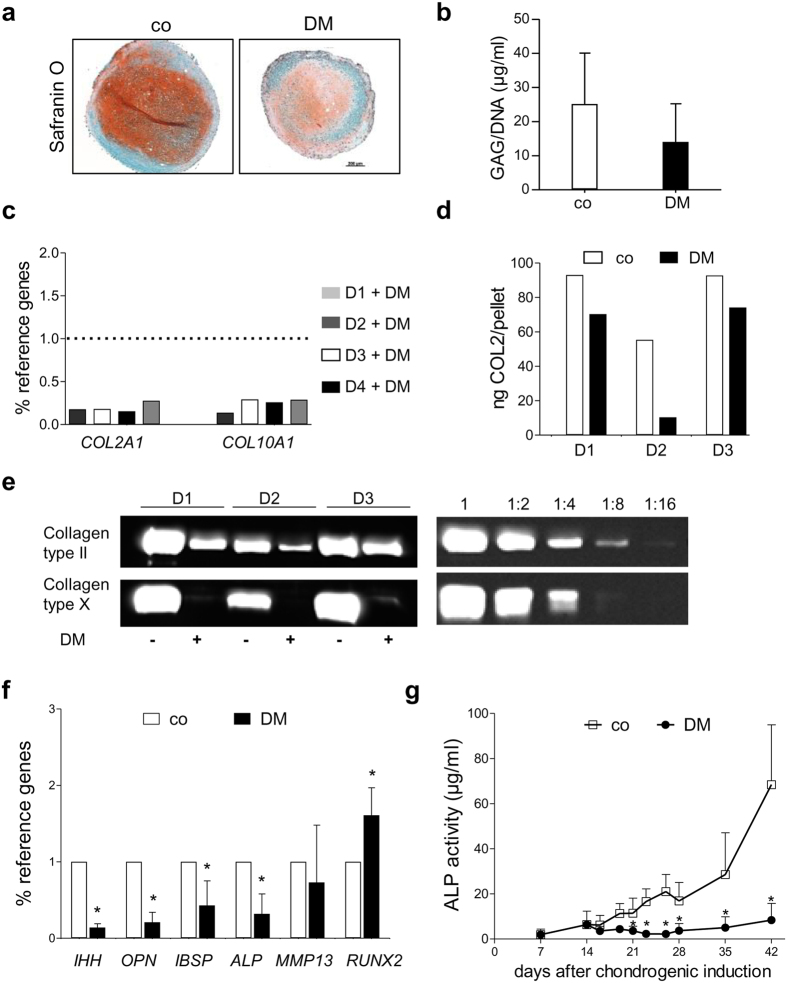
Influence of dorsomorphin treatment on chondrogenic differentiation. Pellets of 4 MSC-populations were cultured in standard chondrogenic medium containing 10 ng/ml TGF-β. From day 14 on, half of the pellets received 0.1% DMSO (co) while the others obtained 10 μM dorsomorphin (DM). After 42 days proteoglycan deposition was determined by (**a**) histological staining of paraffin-embedded sections with Safranin O and (**b**) quantification by DMMB-assay standardized to DNA-content. (**c**) COL2A1 and COL10A1 gene expression at day 42 of pellets from 4 donors (D1-4) versus control set as 1 was measured by qRT-PCR. (**d**) The collagen type II content of day 42 pellets was quantified by ELISA. (**e**) The collagen type II versus collagen type X ratio within the same pellet was analyzed by Western blotting. 64% of the pellet lysate from 3 donors were loaded respectively (left). Dilutions of a standard pellet pool are shown on the right. By cutting the blot, collagen type II and collagen type X content of the same pellet is visualized. Full-length blots are presented in Supplementary Fig. S2. (**f**) Gene expression of IHH, OPN, IBSP, ALP, MMP13 and RUNX2 was tested by qRT-PCR for day 42 pellets. (**g**) At denoted time points, culture supernatant of 5 pellets was harvested, pooled and tested for ALP-activity. Mean values of 4 experiments are shown. Mann–Whitney U test, *p ≤ 0.05 versus control group at the same time point.

Treatment with DM from the chondroblast stage (day 14) on also strongly impaired chondrogenesis, as evident from histological staining (Fig. 6a). While quantitative GAG analysis (Fig. 6b) revealed a trend to less proteoglycan deposition in DM-treated pellets (n = 4 donors) versus controls (p = 0.086), COL2A1 and COL10A1 mRNA levels were about 5-fold lower with DM compared to DMSO (Fig. 6c). Furthermore, more than 10-fold less collagen type II was secreted into culture supernatants at day 28 and 35 (data not shown) and less collagen type II was deposited in pellets at day 42 according to ELISA (Fig. 6d) and Western blotting (Fig. 6e). When compared to serial collagen type II and X dilutions (right part Fig. 6e), collagen type II and collagen type X in the same DM-treated pellet remained more than 4-fold under control conditions (n = 3 donors D1–D3; Fig. 6e) in line with gene expression data. In addition, gene expression of IHH, and of the osteogenic markers OPN, IBSP, and ALP remained significantly lower in the DM group. Remarkably, however, RUNX2, deemed to promote endochondral differentiation, raised (Fig. 6f). This may be explained by the reduced expression of its counter player SOX9 under DM treatment. ALP activity in culture supernatants stayed at the levels when DM treatment was initiated (day 14) and, thus, were significantly lower compared to control cultures from day 21–42 (day 42: reduction by 88%; p < 0.014; Fig. 6g). Furthermore, significantly less MMP13 protein was secreted in the presence of DM according to ELISA (data not shown).

Altogether this demonstrated a considerable contribution of ALK1,2,3,6 stimulation to SOX9 induction and MSC chondrogenesis explaining why inhibition by DM came at the expense of chondrogenic power. Thus, chondrogenesis was considerably delayed by DM treatment and at day 42 cells were in a less mature stage with still lower endochondral markers and collagen type X protein remaining below the detection limit. Overall, chondrogenesis and matrix production ceased under inhibition of ALK1,2,3,6-signalling during chondrogenesis of MSC and no uncoupling of cartilage matrix production from hypertrophy occurred like in AC and achieved under an intermittent PTHrP exposure protocol.

## Discussion

A main restriction in using MSC for cartilage regeneration is their tendency to undergo hypertrophic development and form a mineralizing tissue. By expression of SOX9, RUNX2, IHH, collagen type X, ALP and MMP13^13^,^23^ MSC take the way of endochondral differentiation during *in vitro* chondrogenesis rather than forming stable articular cartilage like expanded AC which maintain their non-hypertrophic phenotype during redifferentiation^13^,^24^. Previous work suggested an active suppression of hypertrophy in articular chondrocytes via secreted negative regulators^25^ like PTHrP and members of the BMP-family^7^,^15^. We previously identified maintained PTHrP expression^16^,^17^ and epigenetic differences like enhanced CpG methylation in the COL10A1 promoter^26^ as mechanisms preventing hypertrophy of AC in redifferentiation culture. Another recent report, however, suggested that enhanced canonical BMP-signalling drives endochondral development of MSC during *in vitro* chondrogenesis indicating that there is yet no full picture of mechanisms responsible for endochondral development of MSC as opposed to chondral redifferentiation of AC.

In here we addressed the differential expression of TGF-β superfamily members and role of Smad1/5/9-signalling in chondral versus endochondral chondrocyte differentiation providing evidence that AC enter into differentiation with a chondrogenic signature, higher mean expression levels of the major TGF-β and BMP members (most importantly BMP4) and with a constitutive pSmad 1/5/9 signalling. In contrast, MSC had an osteochondroprogenitor signature with lower SOX9 but simultaneous RUNX2 expression. In the growth plate, SOX9 keeps RUNX2 expression in check and thereby inhibits progression to prehypertrophy and acquisition of an osteoblastic phenotype^27^. SOX9 protein can directly interact with RUNX2 and repress its activity even in the established osteoblastic lineage^28^. Thus, in search for a means to strengthen SOX9 and suppress RUNX2 activity we herein compared the pSmad signalling between AC and MSC during differentiation and analyzed whether neutralization of pSmad1/5/9 signalling in MSC by DM would sufficiently shift the SOX9/RUNX2 balance towards a dominant SOX9 function. Overall our data demonstrate a strong pro-chondrogenic and SOX9-inducing activity for TGF-β/BMP combinations and an anabolic but not pro-osteogenic role for canonical BMP-signalling in MSC chondrogenesis. Inhibition of pSmad1/5/9 signalling apparently stopped chondrogenesis or decelerated MSC differentiation towards hypertrophy depending on the time of treatment initiation in line with mouse models showing chondrodysplasia and decreased rate of transit through the hypertrophic zone when BMP signalling was diminished^29^. Thus, blocking of canonical BMP signalling apparently was no successful mechanism to install a dominant SOX9 function above RUNX2 since high SOX9 protein levels depended on this pathway.

In favour of a prochondrogenic but not prohypertrophic role of BMP signalling was that AC started into differentiation with a high constitutive BMP-signalling. More cell-autonomous BMP signalling in AC may result in higher Sox9 mRNA (Table 1) and protein levels (Supplementary Fig. S1) at day 0 and faster cartilage matrix deposition in pellet culture compared to MSC. High endogenous BMP4 may also explain why AC can redifferentiate and deposit cartilage matrix in 3D culture even without exogenous TGF-β or BMP support^30^,^31^ while MSC fully rely on the presence of exogenous TGF-β for chondrogenesis^32^. Strong downregulation of BMP4 expression by more than 8-fold during the first 7 days of redifferentiation in AC invites to speculate that cells reduce BMP4 production in the continuous presence of an alternate redifferentiation factor, exogenous TGF-β, for which they may optimize signal transduction by raising TGFBR2 and TGFBR3 and CHRD and CHL2 expression. This contrasted a cell-autonomous up-regulation of BMP4 and BMP7 in MSC during *in vitro* chondrogenesis, demonstrating the fundamentally different regulation of BMP4 and 7 in MSC versus AC. Strong early BMP4 expression and pSmad1/5/9-signalling in chondral AC differentiation but not in endochondral MSC differentiation is difficult to reconcile with a general prohypertrophic role of canonical BMP-signalling in chondrocyte differentiation.

Thus, to further challenge the view of Hellingman *et al.*^5^ that enhanced pSmad1/5/9 during the later phase of MSC chondrogenesis may be responsible for the endochondral rather than chondral development of MSC we provided histology and Western blot data on pSmad2/3 and pSmad1/5/9 levels in AC and MSC in weekly time intervals. A strikingly similar decline of pSmad1/5/9-signalling was seen by histology and Western blotting at later stages of differentiation, demonstrating that the chondral versus endochondral development of AC versus MSC may not be explained by strong differences in canonical pSmad-signalling from day 14 on. Since fine balancing of canonical BMP-signalling not discernible by Western blotting and/or differences in non-canonical signalling may still establish a role for BMPs to drive endochondral development of MSC we boosted BMP-signalling by treatment of MSC with BMP-4/7 or suppressed it by DM treatment from day 14 on.

To simulate MSC chondrogenesis from day 14 on we herein used a heterodimeric BMP-4/7 molecule since heterodimers and homodimers are formed *in vivo* and enhanced effects were ascribed to heterodimeric over homodimeric BMPs^33^. Co-treatment of MSC with TGF-β and BMP-4/7 from day 0 produced additive effects on Sox9 protein levels (Supplementary Fig. S1) and, thus a pro-chondrogenic activity while no shift of MSC differentiation towards hypertrophy or osteogenesis was seen at day 42 after BMP-4/7 treatment from day 14 on. Only induction of ALP and, thus, potential mineralizing activity was significantly accelerated dose-dependently in the presence of BMP-4/7 indicating that the cells enhanced the pace of maturation towards their final differentiation stage. Overall this and the unaltered RUNX2 levels negated a specifically pro-hypertrophic role for BMP-4/7- and pSmad1/5/9-signalling in MSC under chondrogenic conditions.

Karl *et al.* also observed unaltered gene expression of COL2A1 and COL10A1 in MSC when standard chondrogenic medium was supplemented with BMP4 from day 14^34^. Only after withdrawal of TGF-β and dexamethasone from the medium, BMP4 displayed a prohypertrophic activity by enhancing COL10A1 and cell size. They suggested that the antihypertrophic effect of TGF-β and dexamethasone known from growth plate and chicken sternum chondrocytes^35^,^36^,^37^ overrides the pro-hypertrophic effects of BMP4 seen in such models^38^ and a similar explanation may also apply for BMP-4/7 applications in the present study. Since Karl *et al.* did not attempt to quantify BMP effects on IHH, MMP13 and ALP activity described herein, the suppression of IHH and MMP13 as well as the significant acceleration of ALP activation may have escaped their attention. Consequently we argue that enrichment of chondrogenic medium with BMP-4/7 should carefully be decided based on its desired pro-chondrogenic but also undesired pro-mineralizing activity.

During the course of MSC chondrogenesis and AC redifferentiation, pSmad1/5/9-signalling was gradually suppressed. When we accelerated this decline by DM treatment from day 0, MSC chondrogenesis failed herein in line with^5^. Our data imply that insufficient upregulation of SOX9 protein^39^ (drop by over 50% on day 4) at low ALK1,2,3,6-signalling may be a likely reason for this effect. Thus, the lack of chondrogenesis under DM treatment from day 0 may well be explained by insufficient induction of SOX9 due to partial blocking of TFG-β action (Fig. 4a) and blocking of endogenous BMP activity (Table 1). However, DM may also affect chondrogenic power due to interference with additional receptors like the type II receptor ActRIIA^40^ and may even affect TGFBR2 like the structurally similar LDN-193189^41^. Delayed addition of DM from day 14 also strongly reduced collagen type II deposition and appeared to delay chondrogenesis in a way that hypertrophic and osteogenic markers were still significantly lower and no collagen type X protein was yet detected at day 42 by Western blotting^42^. Conclusively this emphasized that an important pro-chondrogenic stimulus was coming from ALK1,2,3,6 downstream signalling at early and late stages of chondrogenesis and its inhibition did not allow an uncoupling of SOX9-driven cartilage matrix production from RUNX2-driven hypertrophy to reach a phenotype similar to AC.

This is in contradiction to Hellingman *et al.*^5^ which described differential pSmad1/5/9 staining between MSC and AC and unaltered COL2A1 and COL10A1 expression at enhanced SOX9 expression under DM treatment at day 35 of differentiation for 3 MSC donor populations. They concluded on a stage-specificity of Smad-signalling during MSC chondrogenesis and reported undisturbed chondrogenesis at selective blocking of hypertrophy under DM from day 14. Other than Hellingman *et al.*^5^, our conclusions on the role of BMP-signalling do not only rely on PCR data and semiquantitative rating of stained tissue sections but are additionally based on Western blot analysis of SOX9 protein and quantitative assessment of collagen type II and collagen type X deposition within the same pellet. Furthermore, in our study, not only one but all five included MSC donor populations underwent strong chondrogenesis allowing us to rate pSmad1/5/9-signalling as an important prochondrogenic stimulus early and later during MSC chondrogenesis without a pronounced prohypertrophic effect in the presence of TGF-β and dexamethasone.

Numerous studies have established that BMP-signalling interacts with the IHH/PTHrP pathway during limb development^7^,^43^ and RUNX2/3-deficient mice do not express IHH in growth plate chondrocytes^44^. In view of the here shown RUNX2 expression and the previously established IHH upregulation during MSC chondrogenesis but not AC redifferentiation^15^ suppression of RUNX2 is an important goal to shift MSC chondrogenesis away from hypertrophy. Silencing of canonical BMP-signalling by DM, however, was here shown to be no adequate means to achieve this goal since SOX9 induction also depended on this pathway. In this respect the recently established capacity of pulsed PTHrP application resulting in strong suppression of ALP activity and IHH expression while even enhancing cartilage matrix deposition^15^,^16^ was much more promising.

In summary, we conclude that the lower SOX9 levels and a RUNX2 expression in MSC, their initially lower mean expression of most TGF-β and BMP molecules and the potentially RUNX2-mediated upregulation of IHH takes MSC into the endochondral route. Silencing of canonical BMP signalling by DM rather decelerated differentiation than specifically suppressing RUNX2 to install a dominance of SOX9 and a lineage shift away from the endochondral pathway since both counterplayers, SOX9 and RUNX2, were inadequately affected by this treatment. Intimate TGF-β/BMP-signalling crosstalk during *in vitro* MSC chondrogenesis and AC redifferentiation with a rather prochondrogenic than proendochondral role for BMPs and pSmad1/5/9-signalling is in line with mouse models showing chondrodysplasia and decreased rate of transit through the hypertrophic zone when BMP signalling was diminished^29^. High early pSmad1/5/9 levels in AC and a strikingly similar decline of pSmad2/3- and pSmad1/5/9-signalling during chondrocyte differentiation negate a main role for canonical BMP-signalling to specifically drive endochondral development during *in vitro* chondrogenesis of MSC but not AC as proposed by Hellingman *et al.*^5^. Future work should address how TGF-β and BMP signalling interacts with the PTHrP/IHH signalling pathway and whether their simultaneous modulation in MSC allows to install a dominant SOX9 function above RUNX2 to shift MSC into the chondral pathway.

## Material and Methods

### Isolation and expansion of MSC

Human bone marrow aspirates were obtained from patients undergoing total hip replacement (n = 14). The study was approved by the local ethics committee (medical faculty of Heidelberg) and written informed consent was obtained from all individuals included in the study, and it was performed in accordance with the 1964 Declaration of Helsinki.

The mononuclear cell fraction was separated from bone marrow aspirates by Ficoll-Paque^TM^ density gradient and seeded into 0.1% gelatin-coated culture flasks in expansion medium composed of DMEM high glucose, 12.5% fetal calf serum (FCS), 2 mM L-glutamine, 1% non-essential amino acids, 50 μM 2-mercapthoethanol (all Gibco, Invitrogen, Karlsruhe, Germany), 100 units/ml penicillin and 100 μg/ml streptomycin (Biochrom AG, Berlin, Germany) and 4 ng/ml basic fibroblast growth factor (bFGF, Active Bioscience, Hamburg, Germany) and maintained at 37 °C in a humidified atmosphere and 6% CO_2_. After 24 h nonadherent cells were removed by washing with PBS.

### Isolation and expansion of AC

Samples of human articular cartilage (n = 13) were obtained from patients undergoing total knee replacement surgery. The study was approved by the local ethics committee (medical faculty of Heidelberg) and written informed consent was obtained from all individuals included in the study, and it was performed in accordance with the 1964 Declaration of Helsinki. Cartilage from regions with no evident degeneration was harvested, minced, and digested overnight with 1.5 mg/ml collagenase B (Roche, Mannheim, Germany) and 0.1 mg/ml hyaluronidase (Merck, Darmstadt, Germany). Chondrocytes were expanded in low-glucose DMEM supplemented with 10% FCS, 100 units/ml of penicillin and 100 μg/ml of streptomycin. In selected experiments AC were expanded in MSC expansion medium.

### Induction of *in vitro* chondrogenesis

MSC from passage 3 and AC from passage 1–3 were resuspended in a chondrogenic medium (DMEM high glucose, 0.1 μM dexamethasone, 0.17 mM ascorbic acid 2-phosphate, 5 μg/ml transferrin, 5 ng/ml sodium selenite, 1 mM sodium pyruvate, 0.35 mM proline, 1.25 mg/ml BSA (all Sigma, Deisenhofen, Germany), 100 units/ml penicillin, 100 μg/ml streptomycin, 5 μg/ml insulin (Sanofi-Aventis, Frankfurt, Germany) and 10 ng/ml TGF-β1 (Humanzyme, Chicago, IL, USA)) and centrifuged (500 g for 5 min) to generate high-density pellets.

Pellets consisting of 5 × 10^5^ cells were cultured for 6 weeks at 37 °C, 6% CO_2_, with a medium change three times a week. Indicated pellet cultures were additionally treated with 10 ng/ml or 100 ng/ml BMP-4/7 heterodimer (R&D Systems, Minneapolis, MN, USA) from day 15–42. Alternatively, the BMP-inhibitor dorsomorphin (10 μM in 0.1% DMSO, ENZO Life Science, Farmingdale, NY, USA) or solvent were added from day 15–42. In one group TGF-β treatment was discontinued after 2 weeks and replaced by 100 ng/ml BMP-4/7 from day 15–42.

### Microarray analysis

Total RNA was isolated from 5 pooled pellets per donor and group. Samples of 5 MSC- and 5 AC-donors were studied at day 0 and day 28 of chondrogenic differentiation. RNA was extracted using RNeasy Mini kit according to manufacturer’s instructions (Qiagen, Hilden, Germany). The microarray analysis (Illumina Human Sentrix, Human Ref_8 v3.0) was performed at the Genomic & Proteomics Core Facility of the German Cancer Research Centre in Heidelberg, Germany. Data analysis was done by normalisation of the signals using the quantile normalisation algorithm without background subtraction and differentially regulated genes were defined by calculating the standard deviation differences of a given probe in a one-by-one comparison of both groups. Genes with a significantly different expression level between groups were annotated using open source Database for Annotation, Visualisation and Integrated Discovery DAVID v6.7^45^,^46^.

### Safranin O staining and collagen type II/X immunohistochemistry

Pellets were fixed in 4% paraformaldehyde (PFA) for 2 hours, dehydrated and paraffin-embedded. Sections (5 μm) were deparaffinized, rehydrated and stained with Safranin O/Fast Green for detection of proteoglycans. Immunohistological staining was performed as described previously^14^. Briefly, sections were pretreated with 2 mg/ml hyaluronidase (Merck) and 1 mg/ml pronase (Roche). PBS containing 5% bovine serum albumin (BSA) was used to block non-specific background. Sections were incubated overnight at 4 °C with a 1:1000 diluted monoclonal mouse anti-human collagen type II antibody (II-4C11, ICN Biomedicals, Aurora, Ohio, USA) or with a mouse anti-human collagen type X antibody (X53, Quartett, Berlin, Germany) in PBS containing 1% BSA. Reactivity was detected using biotinylated goat anti-mouse secondary antibody (1:500; 30 minutes, RT; Dianova, Hamburg, Germany), streptavidin-alkaline phosphatase (30 minutes, 20 °C, Dako, Hamburg, Germany) and fast red (Sigma-Aldrich).

### Immunohistochemistry for pSmad1/5/9

Pellets were paraffin-embedded as described above and sections were treated with 0.3% H_2_O_2_ to block endogenous peroxidase activity and antigen retrieval was performed through incubation with 0.01 M citrate buffer, pH 6.0 and heating in a microwave oven for 10 min. After blocking with 5% skim milk in 1% BSA, sections were incubated overnight with primary antibody for pSmad1/5/9 (1:100, Cell Signalling, #9511) in 1% BSA/PBS. The secondary antibody BrightVision Poly-HRP-Anti Ms/Rb/Rt IgG Biotin-free (Immunologic, Duiven, Netherlands) was used undiluted for 30 min at 20 °C. Antibody complexes were visualized using 50 μl diaminobenzidine-tetra-hydrochloride (DAB, Dako Liquide DAB+Substrate Cromogen System, #K3468) and sections were counterstained with hematoxylin according to standard histology protocols.

### ALP activity

Culture supernatants (five pellets per group) conditioned for 2 days were collected, pooled and incubated with substrate solution (10 mg/ml of p-nitrophenyl phosphate in 0.1 M glycine, 1 mM MgCl_2_ and 1 mM ZnCL_2_, pH 9.6). ALP activity was measured spectrophotometrically at 405/490 nm.

### Proteoglycan content

After chondrogenic differentiation, pellets were predigested in 1 ml of Tris-HCl buffer (0.05 M Tris, 1 mM CaCl_2_, pH 8.0) with 500 μg/ml proteinase K (Roche) over night at 60 °C. 30 μl of adequate diluted samples (dilution in buffer) were mixed with 200 μl 1,9-dimethylmethylene blue (DMMB)-dye solution (3.04 g/l glycine, 2.38 g/l NaCl, 20 mg/l DMMB in distilled water) and absorbance was measured at 530 nm. Standards were prepared from chondroitin sulphate and obtained data were normalized to DNA content^47^.

### Collagen extraction, ELISA and Western Blot

Pellets (n = 1 per donor and group) were digested with pepsin solution [2.5 mg pepsin/ml pepsin buffer (0.5 M acetic acid, 0.2 M NaCl)] for at least 16 hours. The pH was then adjusted to neutral pH 7 with 1 M Tris Base prior to extraction of the collagens with 4.5 M NaCl (overnight at 4 °C, both Roth, Karlsruhe, Germany). After centrifugation, the pellets were resuspended in 400 μl precipitation-buffer (0.1 M Tris Base, 0.4 M NaCl) and the collagens precipitated for 4 hours at −20 °C with 100% ethanol. After centrifugation, the pellets were resuspended in lysis buffer (50 mM Tris, 150 mM NaCl, 1% Triton X-100). The collagen type II content was measured by native type II collagen detection ELISA kit (Chondrex, Redmond, USA) according to manufacturer’s instructions.

For collagen type II and X Western blotting, pellet lysates were separated by denaturing SDS-PAGE and proteins blotted onto a nitrocellulose membrane. The lower part of the membrane was incubated with mouse anti-human collagen type X antibody (Quartett) and the upper part with mouse anti-human collagen type II antibody (ICN Biomedicals). Bands were visualized with peroxidase-coupled goat anti-mouse antibody using the ECL detection system (Roche).

### Detection of Smad and pSmad by Western blotting

Three pellets per donor and group were homogenized mechanically with a Mixer Mill MM 400 (Retsch, Haan, Germany) and resuspended in PhosphoSafe^TM^ Extraction Reagent and 1 mM Pefabloc^®^SC (both Sigma-Aldrich). Cell lysates were centrifuged at 13000 rpm and 4 °C for 20 min and concentration was determined using Bradford assay kit (Sigma-Aldrich). Lysates were separated by SDS-PAGE gel electrophoresis and transferred to a nitrocellulose membrane (GE Healthcare). After blocking for 1 h at 20 °C with 5% skim milk in 0.1% TBST (25 mM Tris/HCl pH 7.4; 145 mM NaCl, 2.7 mM KCl, 0.1% Tween 20) the membrane was incubated over night at 4 °C with primary antibody. The β-Actin (1:10000, GeneTex, GTX26276/18985), pSmad2 (1:250, Cell Signalling, #3108/7), Smad2/3 (1:250, Cell Signalling, #8685/3), pSmad 1/5/9 (1:250, Cell Signalling, #9511/18), Smad1 (1:500, Abcam, ab33902/GR1487581) and Smad5 (1:1000, Abcam, ab40771/YK05017CS) antibody was diluted in 5% skim milk in 0.1% TBST. Next, the blots were washed with 1% TBST and incubated with anti-mouse (Jackson Immuno Research, 115-035-071/29454) or anti-rabbit (Jackson Immuno Research, 111-035-046/27362) secondary antibodies in 5% skim milk in 0.1% TBST for an hour at 20 °C. After washing with 0.1% TBST, the blots were developed with ECL (Roche).

### DNA content

The DNA content of pellets (n = 5 donors) was determined using the Quanti-iT PicoGreen dsDNA kit (Invitrogen) according to the manufacturer’s instructions. For this purpose pellets were harvested at day 42 of chondrogenic induction and pre-digested in 1 ml of Tris-HCl buffer (0.05 M Tris, 1 mM CaCl_2_, pH 8.0) with 500 μg/ml proteinase K (Roche) over night at 60 °C. The next day samples were analyzed by mixing 20 μl of the digested sample with 80 μl TE buffer (200 mM Tris HCl, 20 mM EDTA) and 100 μl PicoGreen solution. Standards were prepared from λ-DNA and fluorescence measurement was carried out at 485/535 nm.

### RNA extraction

Total RNA was isolated from pellets using a standard guanidiniumthiocyanate/phenol extraction protocol (peqGOLDRNAPur^TM^; Peqlab, Erlangen, Germany). mRNA was purified from total RNA with oligo-d(T)-coupled magnetic beads (Dynabeads; Life Technologies). Reverse transcription was performed with 20 ng poly-(A+)-mRNA, Omniscript^TM^ Reverse-Transcriptase (Qiagen), and oligo-d(T) primers. qPCR was performed with the StratageneMx3000P using gene-specific forward and reverse primers (Table 2). Specificity of the PCR products was confirmed by melting curve analysis and agarose gel electrophoresis of PCR products. Gene expression was normalized to reference genes HNRPH1 and CPSF6. The relative difference in expression levels was calculated as described^48^.

### Statistics

Data are presented as mean ± standard deviation. Data were analyzed statistically using Mann-Whitney U-Test and p < 0.05 was considered significant. Data analysis was performed with SPSS for Windows 16.0 (SPSS Inc., Chicago, IL, USA).

## Additional Information

**How to cite this article**: Dexheimer, V. *et al.* Differential expression of TGF-β superfamily members and role of Smad1/5/9-signalling in chondral versus endochondral chondrocyte differentiation. *Sci. Rep.* **6**, 36655; doi: 10.1038/srep36655 (2016).

**Publisher’s note:** Springer Nature remains neutral with regard to jurisdictional claims in published maps and institutional affiliations.

## Supplementary Material

Supplementary Information

## Figures and Tables

**Table 1:** Genes of interest and their regulation in MSC (n = 5) and AC (n = 5) between day 0 and 28 of chondrogenic differentiation

		MSC			AC		
Gene		0d	28d	fold	0d	28d	fold
**Differentiation Markers**							
SOX9	sex determining region Y-box 9	686	3631	**5.3**	1273	2289	1.8
COL2A1	collagen type II	*/*	19762	**304.9**	1960	20028	**10.2**
AGC1	aggrecan 1	*/*	182	**2.8**	353	326	−1.1
COL10A1	collagen type X	*/*	13370	**181.5**	*/*	495	**8.0**
RUNX2	runt-related transcription factor 2	192	411	**2.1**	*/*	145	**2.2**
SPP1, OPN	osteopontin	110	10721	**97.7**	*/*	132	1.9
ALP	alkaline phosphatase	553	9304	**16.8**	1118	122	**−9.2**
MMP 13	matrix metalloproteinase 13	/	233	**3.8**	*/*	*/*	*/*
Receptors*							
ACVR-IB	activin A receptor, type 1B (ALK4)	333	225	−1.5	335	193	−1.7
ACVR-I	activin A receptor, type 1 (ALK2)	802	925	1.2	1368	1268	−1.1
BMPR2	BMP receptor type 2	253	375	1.5	293	376	1.3
*TGFBR2*	TGF-β receptor type 2	2566	793	**−3.2**	992	2593	**2.6**
*TGFBR3*	TGF-β receptor type 3	1027	437	**−2.4**	565	1943	**3.4**
**Growth Factors**							
*BMP2*	bone morphogenetic protein 2	119	476	**4.0**	376	196	−1.9
BMP3	bone morphogenetic protein 3	/	/	/	/	/	/
*BMP4*	bone morphogenetic protein 4	232	594	**2.6**	3227	383	**−8.4**
BMP5	bone morphogenetic protein 5	183	95	−1.9	/	/	/
*BMP6*	bone morphogenetic protein 6	87	81	−1.1	108	/	/
*BMP7*	bone morphogenetic protein 7	/	/	/	/	/	/
GDF5	growth differentiation factor 5	154	/	**−2.0**	150	236	1.6
TGFB1	transforming growth factor beta 1	/	/	/	/	/	/
TGFB2	transforming growth factor beta 2	89	86	1.0	142	90	−1.6
TGFB3	transforming growth factor beta 3	266	468	1.8	433	265	−1.6
**Inhibitors**							
*CHL2*	chordin-like 2	/	/	/	219	142	−1.5
*CHRD*	chordin	/	86	/	87	106	1.2
*FST*	follistatin	3570	3031	−1.2	6962	1185	**−5.9**
FSTL1	follistatin-like 1	1368	1805	1.3	4418	3277	−1.4
FSTL3	follistatin-like 3	140	598	**4.2**	250	451	1.8
FSTL4	follistatin-like 4	/	/	/	/	/	/
FSTL5	follistatin-like 5	/	/	/	/	/	/
GREM2	gremlin 2	200	103	−1.9	/	/	/
NOG	noggin	/	/	/	/	/	/
SOST	sclerostin	/	/	/	/	/	/
*only molecules above background are shown **bold **= regulation ≥2-fold *italics *= also tested by real-time PCR / = mean value of datasets from 5 donors below background (80)							

**Table 2:** List of oligonucleotides used for quantitative RT-PCR analysis.

Gene		Forward primer	Reverse primer
ALP	alkaline phosphatase	5′-caccaacgtggctaagaatg-3′	5′-atctccagcctggtctcctc-3′
BMP4	bone morphogenetic protein 4	5′-ggatctttaccggcttcagtc-3′	5′-cctgggatgttctccagatg-3′
BMP7	bone morphogenetic protein 7	5′-ccagaaccgctccaagac-3′	5′-gttggtggcgttcatgtag-3′
CHRD	chordin	5′-gtggctcagaacaaggcact-3′	5′-ctccaggtccttcaccacac-3′
CHL2	chordin-like 2	5′-ccaagcccagacaacctg-3′	5′-gggccaggtacttcacctct-3′
COL2A1	collagen type II	5′-tggcctgagacagcatgac-3′	5′-agtgttgggagccagattgt-3′
COL10A1	collagen type X	5′-ccctttttgctgctagtatcc-3′	5′-ctgttgtccaggttttcctggcac-3′
*CPSF6*	cleavage and polyadenylation specificity factor subunit 6	5′-aagattgccttcatggaattgag-3′	5′-tcgtgatctactatggtccctctct-3′
FST	follistatin	5′-tctgccagttcatggagga-3′	5′-tccttgctcagttcggtctt-3′
*HNRPH1*	heterogeneous nuclear ribonucleoprotein H1	5′-gatgtagcaaggaagaaattgttcag-3′	5′-caccggcaatgttatcccat-3′
IBSP	integrin binding sialoprotein	5′-cagggcagtagtgactcatcc-3′	5′-tcgattcttcattgttttctcct-3′
MMP13	matrix metalloproteinase 13	5′-ctggagatatgatgatactaac-3′	5′-cacgcatagtcatatagatact-3′
OPN	osteopontin	5′-tccaagtaagtccaacgaaag-3′	5′-tctacaaccagcatatcttca-3′
TGFBR2	transforming growth factor beta receptor 2	5′-atggaggcccagaaagatg-3′	5′-gactgcaccgttgttgtcag-3′
TGFBR3	transforming growth factor beta receptor 3	5′-ggcacacactttgttttggag-3′	5′-aagggctggaacctgtatca-3′

italic: reference genes.
