# Supplementary material for: Differential expression of TGF-β superfamily members and role of Smad1/5/9-signalling in chondral versus endochondral chondrocyte differentiation
Source: Sci Rep. 2016 Nov 16;6:36655. doi: 10.1038/srep36655 (PMC5111074; doi:10.1038/srep36655)
Supplement: Supplementary Information [file srep36655-s1.doc]

**Differential expression of TGF-β superfamily members and role of Smad1/5/9-signalling in chondral versus endochondral chondrocyte differentiation**

Verena Dexheimer^1^, Jessica Gabler^1^, Katharina Bomans^1^, Tanja Sims^1^, Georg Omlor^2^ and Wiltrud Richter^1*^

^1^Research Centre for Experimental Orthopaedics, Orthopaedic University Hospital Heidelberg, Heidelberg, Germany

^2^Department of Orthopaedic and Trauma Surgery, University Hospital Heidelberg, Heidelberg, Germany

Addresses of the authors

Verena Dexheimer: verena.dexheimer@googlemail.com

Jessica Gabler: jessica.gabler@med.uni-heidelberg.de

Katharina Bomans: katharina.bomans@med.uni-heidelberg.de

Tanja Sims: tanja.sims@med.uni-heidelberg.de

Georg Omlor: georg.omlor@med.uni-heidelberg.de

Wiltrud Richter: wiltrud.richter@med.uni-heidelberg.de

*Address for correspondence:

Prof. Dr. Wiltrud Richter

Research Centre for Experimental Orthopaedics

Orthopaedic University Hospital Heidelberg

Schlierbacher Landstrasse 200a

69118 Heidelberg

Germany

Phone: +49 6221 56 29254

Fax: +49 6221 56 29288

Email: wiltrud.richter@med.uni-heidelberg.de

Authors disclose any potential conflict of interest regarding to this work.


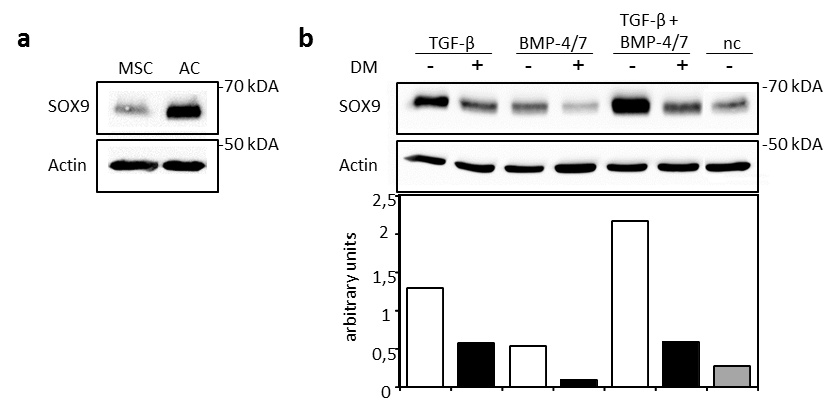


Supplementary Fig. S1. SOX9 protein in MSC and AC and the effect of dorsomorphin on SOX9 protein levels. MSC and AC were expanded up to passage 3 and 5x10^5^ cells were immediately harvested at day 0 (a) or pellets consisting of 5x10^5^ MSC were stimulated for 4 days (b) in chondrogenic basal medium (nc). Half of the pellets were treated with 10 µM dorsomorphin (DM) or received the solvent DMSO (0.1%). Additionally pellets were treated with 10 ng/ml TGF-ß, 100 ng/ml BMP-4/7 or 10 ng/ml TGF-ß + 100 ng/ml BMP-4/7. SOX9 protein was detected by immunoblotting and quantified densitometrically.


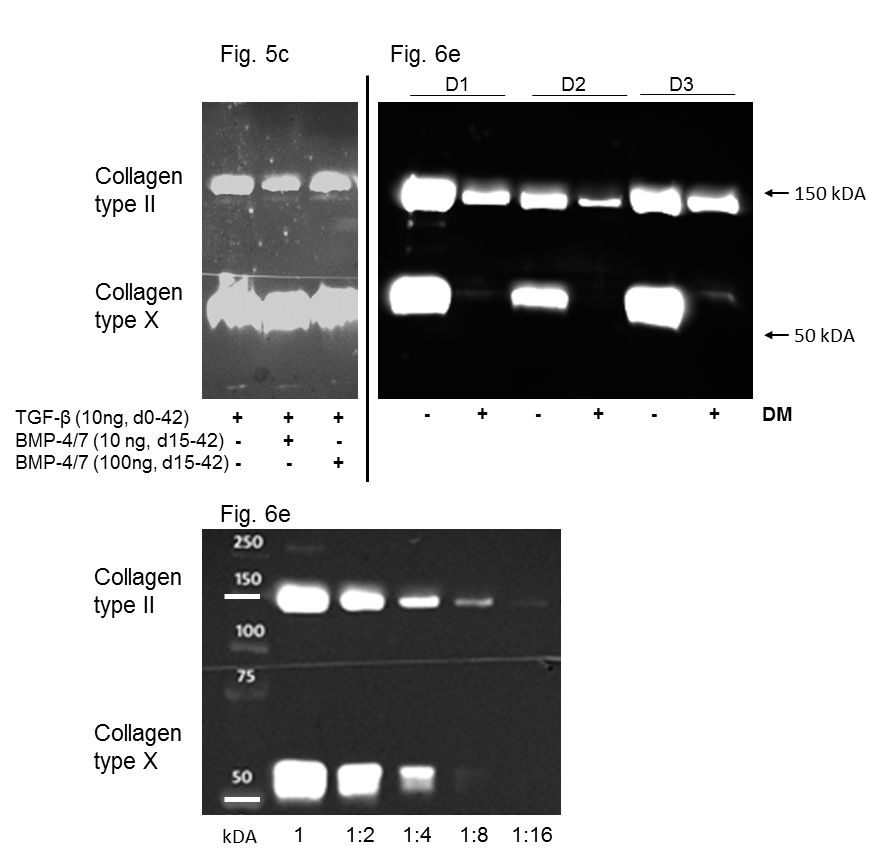


Supplementary Fig. S2. Full-length blots of cropped version used in Fig. 5c and Fig. 6e.
